# Microcatheter-Assisted Trabeculotomy Combined With Deep Sclerectomy and Trabeculectomy in Young to Middle-Aged Adults With Advanced Primary Open-Angle Glaucoma: 1-Year Result

**DOI:** 10.3389/fmed.2021.712332

**Published:** 2021-09-03

**Authors:** Hengli Zhang, Xiaowei Yan, Fan Li, Lihua Ma, Yulei Geng, Kexin Jiao, Guangxian Tang

**Affiliations:** Department of Ophthalmology, Shijiazhuang People's Hospital, Shijiazhuang, China

**Keywords:** primary open angle glaucoma, microcatheter, trabeculotomy, deep sclerectomy, trabeculectomy

## Abstract

**Objective:** We aimed to evaluate the safety and clinical efficacy of *ab externo* microcatheter-assisted trabeculotomy combined with deep sclerectomy and trabeculectomy (MATT-DS-Trab) in the surgical management of advanced primary open-angle glaucoma (POAG).

**Methods:** According to the inclusion criteria, we retrospectively collected and analyzed 37 POAG cases in advanced stage who received MATT-DS-Trab. The intraocular pressure (IOP), best corrected visual acuity (BCVA), use of anti-glaucoma drugs, shape of the filtering bleb, size of the scleral lake, complications, and the surgical success rate were recorded.

**Results:** The mean IOP was 37.50 ± 8.11 mmHg before the operation, while it depleted to 10.08 ± 2.01 and 11.43 ± 2.07 mmHg at 1 week and 12 months after the operation, respectively (both *P* < 0.001 compared to preoperative IOP). From none to two kinds of anti-glaucoma drugs were used 12 months after surgery on the patients, which were significantly reduced compared with that preoperatively (*P* < 0.001). An L-type filtering bleb was the main form at all time points after the operation. At 12 months following surgery, an F-type filtering bleb accounted for 5.41% and no E-type filtering bleb was recorded. The length and height of the scleral lake shrunk with time, but there was no statistical significance (*P* > 0.05). Also, there was no correlation between the size of the scleral pool and the IOP (*P* > 0.05). At 12 months after the operation, the complete success rates were 94.59, 83.78, and 72.97% according to standards A (≤18 mmHg), B (≤15 mmHg), and C (≤12 mmHg), respectively. Intraoperative complications were mainly anterior chamber hemorrhage, and no complications related to the filtration bleb were observed after the operation.

**Conclusion:** Based on multichannel mechanisms, MATT-DS-Trab is able to effectively reduce IOP in advanced POAG patients, with few serious complications and a high success rate.

## Introduction

Primary open-angle glaucoma (POAG) often causes blindness. Trabeculectomy is a classic operation used to treat glaucoma and is considered one of the primary surgical treatments for POAG. However, the success rates were 86.5, 69.3, and 53.1% at 1, 3, and 5 years after surgery, respectively, after which the failure rate increased by an additional 10% each year (1–3). The primary cause of failure was scarring of the filtering area. *Ab externo* microcatheter-assisted trabeculotomy (MATT) is performed by inserting an iTrack laser microcapsule into the Schlemm's canal (SC) through an incision in the outer wall of 360°. We then incised the inner wall of the SC and the trabecular meshwork (TM). This may promote drainage of aqueous humor and reduce intraocular pressure (IOP) when combined with accurate surgical positioning. The success rate of congenital glaucoma surgery (IOP < 21 mmHg) by MATT could be as high as 83–91% after 6–12 months (4–7), and the 2-year complete success rate may be as high as 67% (IOP <18 mmHg) (8). Studies have shown that trabeculotomy for POAG in adults is not as effective as in the case of congenital glaucoma in lowering the IOP (9, 10). But the investigation by Grover et al. presented that circumferential *ab interno* trabeculotomy in adults with POAG is effective; the mean IOP ranged from 15.5 to 16.2 mmHg with 1.7 glaucoma drugs 12 months after surgery (10). Grant believed that most of the outflow resistance of the aqueous humor lies in the SC–TM complex. Trabeculotomy can reduce aqueous humor resistance by 75% (11). However, some studies have investigated that trabeculotomy can only eliminate 40–50% of outflow resistance when the IOP perfusion is low (12, 13). Trabeculotomy is more suited to treat patients with POAG in the early and moderate stages who have not adapted to glaucoma filtering surgery and whose IOP values do not need to be reduced to <15 mmHg (14).

However, most patients with POAG in China are already in the late stage when diagnosed, and the IOP remains high even after the maximum dose of drug treatment has been administered. Some patients are likely to develop scarring after trabeculectomy, and their IOP must be reduced to protect the remaining visual function (15). For such patients, it is important to discover a safe and effective way to reduce the IOP. In the present study, we describe a series of Chinese patients with advanced POAG who underwent microcatheter-assisted trabeculotomy combined with deep sclerectomy and trabeculectomy (MATT-DS-Trab). The operation aims to reduce the IOP stably to values within the target range based on multichannel aqueous humor drainage mechanisms and to eventually improve long-term efficacy and reduce postoperative complications.

## Materials and Methods

### Retrospective Case Series

We retrospectively collected and analyzed data from 37 patients with advanced POAG who received MATT-DS-Trab between September 2018 and April 2019 at Shijiazhuang People's Hospital. The study included 26 men and 11 women. Their ages ranged between 19 and 57 years (37.46 ± 12.08). The preoperative IOP was 37.50 ± 8.11 mmHg despite the patients receiving the maximum dose of IOP-controlling drugs. All operations were performed by the same surgeon in the same manner. All patients were followed up for longer than 12 months after surgery. This study was approved by the ethics committee of Shijiazhuang People's Hospital. All patients were informed and signed a surgery consent form.

### Diagnosis and Inclusion and Exclusion Criteria

#### Diagnostic and Inclusion Criteria

Gonioscopy was performed for patients with open-angle glaucoma and a clear structure, while fundus stereography was for patients with glaucoma optic neuropathy (16), cup/disc (C/D) > 0.8. Patients' signs and symptoms coincided with the diagnostic criteria of advanced POAG according to the Hodapp–Anderson–Parris staging method and the Advanced Glaucoma Intervention Study (AGIS) system rating (17). The inclusion criteria were as follows: (1) patients older than 18 years (based on PubMed's MeSH definition for young middle-aged adults) (18) who did not have a history of previous eye surgery; (2) the patient's IOP was still uncontrolled in the target range (≥21 mmHg) despite taking the maximum tolerable number of anti-glaucoma medications (three or more) or a fluctuation of more than 8 mmHg during a 24-h IOP measurement and the progression of visual field loss over time; and (3) patients who could not tolerate or had serious adverse effects when using anti-glaucoma drugs.

#### Exclusion Criteria

We excluded the following: (1) patients whose fundus could not be observed due to refractive ocular diseases; (2) patients who had undergone eye surgeries such as cataract surgery, corneal surgery, etc.; (3) patients with secondary glaucoma, e.g., neovascular glaucoma; (4) patients with peripheral anterior synechiae (PAS); (5) monocular patients; (6) patients with severe systemic or mental conditions; and (7) pregnant women.

### Surgical Procedure

All surgeries were conducted under topical anesthesia with proparacaine hydrochloride eye drops and subconjunctival anesthesia in the surgical area with 0.2 ml of a 2% lidocaine solution. A fornix-based conjunctival flap was created and a 5 × 5-mm superficial scleral flap one-third as thick as the sclera, extending ~1 mm into the clear cornea, was excised. A piece of sponge soaked in 0.4 mg/ml mitomycin (MMC) was applied under the conjunctiva and scleral flap for 3–4 min, followed by thorough washing. An ~4 × 4-mm-deep sclera was then created, leaving a margin of 0.5 mm on each side, along with a thinner layer of deep sclera covering the choroid. The color of the pigmentation in the choroid tissue was visible in the sclera bed. Paracentesis was then performed at a depth of 0.5 mm inside the transparent corneal limbus at the temporal side, and an appropriate amount of the aqueous humor was released to allow the IOP to return to a value within the normal range. Subsequently, the SC in front of the scleral spur was examined and opened, and the end of the SC was dilated by viscoelastic using a specially designed needle. The illuminated microcatheter (iTrack 250A, iScience Interventional, Menlo Park, CA, USA) was inserted into the SC and threaded circumferentially around it. In four cases, the microcatheter encountered some resistance. When this occurred, the microcatheter was pulled out and threaded again in the opposite direction. An appropriate amount of viscoelastic material was again injected into the anterior chamber through the puncture opening. Then, both exposed ends of the microcatheter were grasped and pulled in opposite directions, thereby conducting the trabeculotomy. The remainder of the trabeculectomy procedure has been described previously (19). The deep scleral flap and a 1.5 × 3-mm portion of the trabeculum were excised, and a peripheral iridectomy was then performed. The two posterior corners of the superficial scleral flap were fixed using a 10–0 nylon suture under moderate tension, and two releasable sutures were made tightly on the vertical incisions of both sides of the superficial scleral flap to temporarily fix the scleral flap firmly in place. Gentle anterior chamber irrigation was performed *via* paracentesis in cases of significant hyphema. The anterior chamber was subsequently rebuilt and the filtering function was evaluated. The conjunctiva was closed using a 10–0 nylon suture. Finally, the IOP was elevated to a normal level. All surgeries were performed by a single experienced glaucoma surgeon (GX Tang).

### Observational Indicators

The following parameters were examined and analyzed before surgery and at 1, 7, and 14 days and at 1, 3, 6, and 12 months after surgery. The BCVA was measured using the Snellen chart and the results described using the logarithmic minimum angle of resolution (logMAR). IOP was measured using a calibrated Goldman applanation tonometer. Slit lamp microscopy, gonioscopy (a single-mirror Gonio diagnostic lens), ultrasound biomicroscopy (UBM) (300, Meda Co., Ltd., Tianjin, China), stereoscopic optic disc photography (Kowa Nonmyd WX 3D, Tokyo, Japan), the number of anti-glaucoma medications, surgical success rate, and the occurrence of complications were observed. We also assessed the head of the optic nerve and the visual field (VF) using optical coherence tomography (Heidelberg, Germany) and a Humphrey-750i Field Analyzer (Carl Zeiss Meditec, Oberkochen, Germany). All examinations were performed by experienced ophthalmologists and technicians.

### Postoperative Management

The subjects were prescribed tobramycin dexamethasone drops four times a day and tobramycin dexamethasone ointment once a day after the procedure. The medication frequency gradually subsided following relief from ocular inflammatory reaction during the follow-up period. A 2% pilocarpine solution was given four times a day for 3 months to prevent PAS (20). Postoperative gonioscopy was performed at each hospital visit.

We obtained the images using UBM and examined the area at a depth of 5 mm with a probe frequency of 50 MHz and observed the scope displayed on the monitor (8 × 5.5 mm). Additional observations with UBM were conducted if the IOP was >18 mmHg. All patients underwent a scan of the surgical area and an evaluation according to the UBM procedure and calculation method described in a previous study (21). Quantitative observation included measurements of the scleral lake size, maximum anteroposterior length (MAPL) of the longitudinal scan, and maximum height (MH). The filtration blebs were classified as L-type (low-reflective), H-type (high-reflective), E-type (encapsulated), and F-type (flattened) in accordance with the methods used in a previous study and the parameters assessed with UBM (22). Morphological changes in the filtration bleb and intrascleral lake were measured 1, 3, 6, and 12 months postoperatively using UBM.

### Surgical Success Rate

Complete success was defined as IOP values ranging between 5 and 18 mmHg with a reduction of at least 30% from the baseline IOP (23). Postsurgical IOP ≤18 mmHg was defined as criterion A, ≤15 mmHg as criterion B, and ≤12 mmHg as criterion C. These criteria had to be fulfilled without the use of anti-glaucoma drugs. Qualified success referred to an IOP that fulfilled the above-mentioned criteria after the topical application of anti-glaucoma medications. Failure was defined as two consecutive determinations of IOP that exceeded the aforementioned IOP values after topical application of three or more anti-glaucoma drugs.

### Statistical Analysis

SPSS 19.0 software (IBM, Chicago, IL, USA) was used for all data analyses. The Shapiro–Wilk test was used to test normality, and parametric or non-parametric tests were applied accordingly. Descriptive data for numeric variables were presented as the mean ± SD, as medians and interquartile range for continuous variables, or as *n* (%) for categorical variables. Preoperative and postoperative IOP values were compared using a paired *t*-test. The Wilcoxon paired signed-rank test was used to compare BCVA and the number of drugs used before and after surgery. The Kruskal–Wallis *H* test was conducted to compare changes in the number of drugs at different time points after surgery. Filtration bleb morphologies were assessed using Fisher's exact test. MAPL and MH at different time points after surgery were compared using parametric one-way analysis of variance (ANOVA). Pearson's correlation coefficient or Spearman's correlation coefficient was used to evaluate the correlation between IOP and scleral lake parameters. Kaplan–Meier curve analysis was performed to determine the cumulative probability of complete surgical success. Statistical significance was set at *P* < 0.05.

## Results

### Characteristics of Patients

The mean follow-up time was 17.20 ± 3.4 months (13–25 months). Table 1 shows the preoperative information for all patients.

**Table 1:** Baseline parameters for the subjects.

**Parameter**	**Results**
Subjects (*n*, %)	37 (100.00)
Eyes (*n*, %)	37 (100.00)
**Gender**
Male (*n*, %)	26 (70.27)
Female (*n*, %)	11 (29.73)
**Age (years)**
<30 years	12 (32.43)
30–40 years (included 30 and 40 years)	10 (27.03)
40–50 years (included 50 years)	9 (24.32)
>50 years	6 (16.22)
**C/D**
0.9	17 (45.95)
1.0	20 (54.05)
**MD (dB)**
Mean ± SD	−20.62 ± 7.63
Minimum, maximum	−30.72, −13.08
**AL (mm)**
Mean ± SD	23.43 ± 0.99
Minimum, maximum	22.66, 24.06
**Refractive error (D)**
Mean ± SD	−0.23 ± 1.27
Minimum, maximum	−2.25, +2
**Number of medications**
3	9 (24.32)
4	25 (67.57)
5	3 (8.11)

*C/D, cup/disc; MD, mean deviation; SD, standard deviation; AL, axial length; D, diopter*.

### Best Corrected Visual Acuity

The Shapiro–Wilk test showed that the BCVA (LogMAR) was statistically significant both before and after surgery (all *P* < 0.001) and did not conform to normal distribution. The Wilcoxon paired signed-rank test did not reveal a significant difference in the BCVA (LogMAR) before and 12 months after the procedure (*Z* = −0.834, *P* = 0.404) (Table 2).

**Table 2:** Changes in the BCVA (LogMAR) before and after surgery.

**Time point**	**Median**	**Q1**	**Q3**	***Z***	***P*^a^**
Preoperative	0.11	0.00	0.19	–	–
12 months postoperative	0.12	0.00	0.22	−0.834	0.404

*BCVA, best corrected visual acuity; LogMAR, logarithmic minimum angle of resolution; Q1, lower quartile; Q3, upper quartile*.

a *Wilcoxon paired signed-rank test was used before and after surgery*.

### Intraocular Pressure

The mean IOP before surgery was 37.50 ± 8.11 mmHg. The mean IOP and the corresponding pressure decrease at 1 and 2 weeks and at 1, 3, 6, and 12 months after surgery are shown in Table 3. Compared with the preoperative IOP, the postoperative IOP at 1 and 2 weeks and at 1, 3, 6, and 12 months diminished by 69.91, 69.67, 68.59, 67.93, 68.06, and 68.14%, respectively. There were significant differences in the IOP at all time points before and after surgery (all *P* < 0.001).

**Table 3:** Changes in IOP before and after surgery.

**Time point**	***n***	**IOP (mmHg)**		**IOP decrease from baseline**		***t***	***P*^a^**
		**Mean ± SD**	**95% CI**	**Mean ± SD**	**95% CI**		
Preoperative	37	37.50 ± 8.11	33.78–39.19			–	–
**Postoperative**
1 week	37	10.08 ± 2.01	9.60–10.94	26.22 ± 8.12	23.51–28.92	19.644	<0.001
2 weeks	37	10.58 ± 1.76	9.99–11.20	26.13 ± 8.50	23.25–29.00	18.449	<0.001
1 month	37	11.44 ± 1.71	10.42–11.58	25.72 ± 8.38	22.89–28.56	18.412	<0.001
3 months	37	11.57 ± 2.20	10.50–11.99	25.48 ± 8.49	22.60–28.35	18.006	<0.001
6 months	37	11.39 ± 2.10	10.79–12.24	25.52 ± 8.62	22.61–28.44	17.390	<0.001
12 months	37	11.43 ± 2.07	10.82–12.27	25.55 ± 8.36	22.98–28.65	18.011	<0.001

*IOP, intraocular pressure; CI, confidence interval*.

a *Paired t-test was conducted before and after surgery*.

### Medications

Patients required three to five kinds of anti-glaucoma medication before the operation and none or only one medication at 3 and 6 months after surgery; the number of anti-glaucoma medications was significantly less than that used preoperatively (*P* < 0.001). There was also a marked reduction in the number of medications used at 12 months postoperatively (from none to two types) compared to the number used preoperatively (*P* < 0.001) (Table 4).

**Table 4:** Number of medications before surgery and at 3, 6, and 12 months after surgery.

**Time point**	**No. of medication, median (range)**	***Z***	***P*^a^**
Preoperative	4 (3–5)		
**Postoperative**
3 months	0 (0–1)	−5.50	<0.001
6 months	0 (0–1)	−5.50	<0.001
12 months	0 (0–2)	−5.476	<0.001

a *Wilcoxon paired signed-rank test between baseline and post-baseline values*.

### Changes in the Morphology of the Filtering Bleb and Sclera Pool Before and After Operation

#### Morphology of the Filtering Bleb and the Appearance of Anterior Chamber Angle

**Figure 1:**
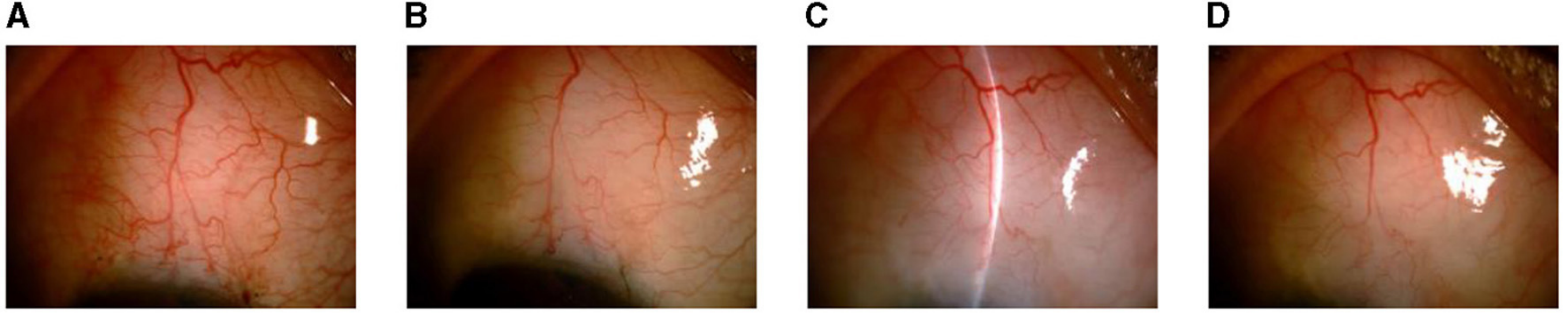
Changes in the filtering blebs in the slit lamp images at different time points after surgery. **(A)** At 1 month after surgery. **(B)** At 3 months after surgery. **(C)** At 6 months after surgery. **(D)** At 12 months after surgery.

**Figure 2:**
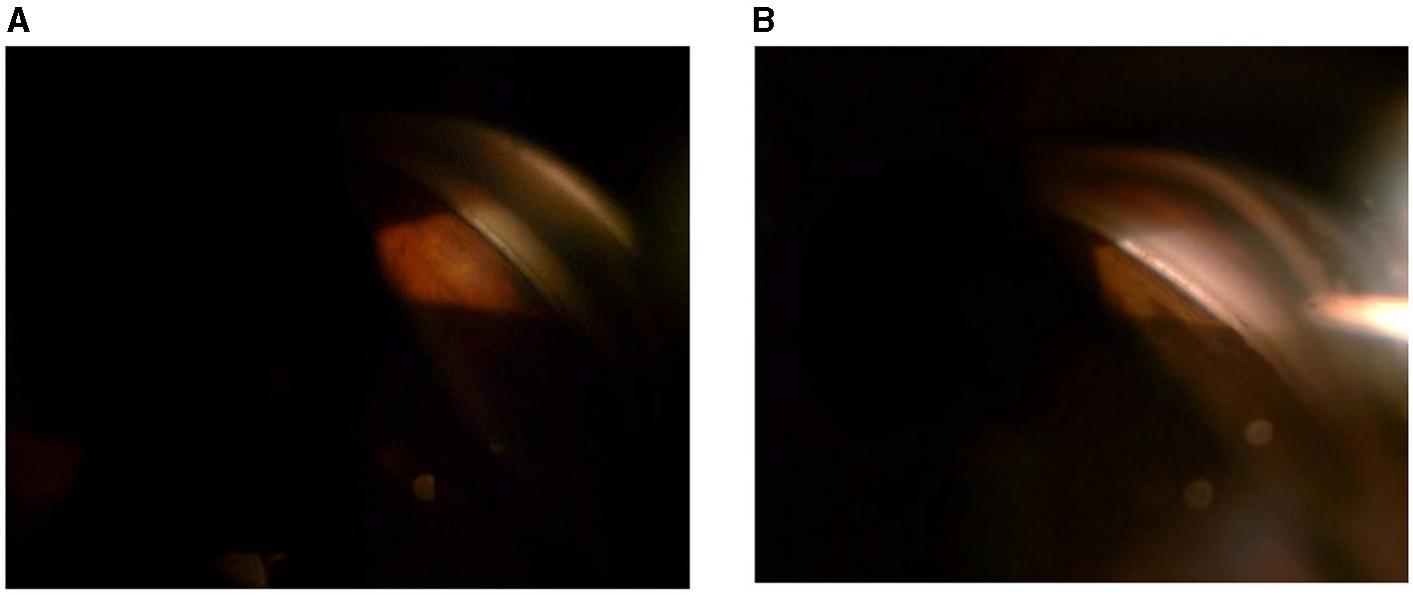
**(A,B)** Appearance of the anterior chamber angle after microcatheter-assisted trabeculotomy combined with deep sclerectomy and trabeculectomy (MATT-DS-Trab) at 1 month **(A)** and at 12 months **(B)** after surgery.

At 1, 3, 6, and 12 months after the operation, the L-type filtering bleb accounted for 97.30, 97.30, 97.30, and 94.59% and the F-type accounted for 0.00, 2.70, 2.70, and 5.41% of the blebs, respectively. One month after the operation, the proportion of H-type filtering blebs was 2.70%. No E-type filtering blebs were observed during the entire follow-up period. There was no statistical difference between the groups (*F* = 4.98, *P* = 0.55) (Table 5 and Figure 1). The appearance of the anterior chamber angle after surgery is presented in Figure 2.

**Table 5:** Changes in the filtering bleb morphology after surgery.

**Time point**	**Filtering bleb morphology**				***F***	***P*^a^**
	**L (*n*)**	**H (*n*)**	**E (*n*)**	**F (*n*)**		
1 month	36	1	0	0		
3 months	36	0	0	1		
6 months	36	0	0	1		
12 months	35	0	0	2		
					4.98	0.55

*L, low-reflective type; H, high-reflective type; E, encapsulated type; F, flattened type*.

a *Fisher's exact test was used*.

#### Sclera Pool

**Figure 3:**
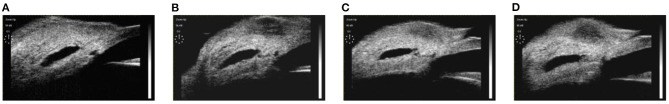
Changes in the scleral reservoirs in ultrasound biomicroscopy (UBM) images at different time points after surgery. **(A)** At 1 month after surgery. **(B)** At 3 months after surgery. **(C)** At 6 months after surgery. **(D)** At 12 months after surgery.

Hypoechoic images were taken under the scleral flap at different time points after surgery. The length and the height of the scleral pool are shown in Table 6. The length and height of the scleral cistern tended to shrink after a longer observation time had elapsed, but the difference was not significant (MAPL: *F* = 1.13, *P* = 0.34; MH: *F* = 1.24, *P* = 0.30) (Table 6 and Figure 3). No significant correlation was observed between IOP and the scleral pool length or height during the entirety of the follow-up period (Table 7).

**Table 6:** Changes in the scleral lake after surgery.

**Time point**	**MAPL (μm)**		**MH (μm)**	
	**Mean ± SD**	**95% CI**	**Mean ± SD**	**95% CI**
1 month	3,450.30 ± 495.77	3,285.00–3,615.60	711.81 ± 106.33	676.36–747.26
3 months	3,360.62 ± 482.87	3,199.63–3,521.62	688.95 ± 102.89	654.64–723.25
6 months	3,336.43 ± 479.37	3,176.60–3,496.26	676.22 ± 100.99	642.55–709.89
12 months	3,246.03 ± 466.46	3,090.50–3,401.55	669.03 ± 99.90	635.72–702.34
*F*	1.13	–	1.24	–
*P* ^a^	0.34	–	0.30	–

*MAPL, maximum anteroposterior length; MH, maximum height; CI, confidence interval*.

a *ANOVA (parametric)*.

**Table 7:** Correlations between MAPL, MH, and IOP after surgery.

**Correlation with IOP (mmHg)**								
	**1 month**		**3 months**		**6 months**		**12 months**	
	***r***	***P*^a^**	***r***	***P*^a^**	***r***	***P*^a^**	***r***	***P*^a^**
MAPL (μm)	−0.173	0.306	−0.154	0.364	−0.269	0.107	−0.203	0.228
MH (μm)	−0.223	0.184	−0.193	0.253	−0.147	0.387	−0.184	0.276

*MAPL, maximum anteroposterior length; MH, maximum height; IOP, intraocular pressure*.

a *Spearman's rank correlation coefficient*.

### Intraoperative, Postoperative Complications, and Follow-Up

During the operation, 33 eyes were simultaneously threaded circumferentially with the illuminated microcatheter tip. Resistance was encountered in four eyes during the threading process. After withdrawing from the original route, we successfully completed the procedure in the reverse direction. The rate of success was 100%. Intraoperative complications, such as hyphema in the anterior chamber, occurred in all patients, but blood was absorbed within 2 weeks following the operation. During the operation, one eye experienced Descemet's layer detachment with a range of 2 × 3 mm at six points below the cornea. Sterile gas was administered in the anterior chamber after the operation and the patient recovered well.

One eye developed a shallow anterior chamber and a relatively low IOP (9 mmHg) 1 week postoperatively, but the eye was left untreated and recovered after 1 month. Two weeks after surgery, one eye had a flat choroidal detachment, and the IOP was maintained at 7 mmHg. Hypotony maculopathy did not occur, and no special treatment was given except for regular follow-up in the clinic.

In addition, the IOP increased to 42 mmHg in one eye. The ciliary body band was observed with gonioscopy, and UBM images showed hyperechoic reflection under the filtering bleb and scleral flap 3 weeks postoperatively. Interestingly, after the scleral flap suture was released by laser under a slit lamp, the filtering bleb diffused and swelled and the IOP dropped to 9 mmHg. At the last follow-up, the IOP of the patient was 11 mmHg. In addition, four eyes experienced PAS, among which three had synechia under 90° (IOP = 11–13 mmHg) and one underwent 180° adhesion (the IOP fluctuated between 13 and 15 mmHg); no special treatment was administered. The IOP was 21 mmHg in one eye and abated to 14 mmHg at 3 months postoperatively after one anti-glaucoma drug was administered. During the follow-up period, the IOP of another eye peaked at 22 mmHg during a 24-h IOP measurement, with a fluctuation of 11.3 mmHg. The IOP decreased to 13.7 mmHg after treatment with two topical medications. At the last follow-up, no complications (e.g., a thin-walled filtering bleb or filtering bleb leakage) were observed.

### Surgical Success Rates

**Figure 4:**
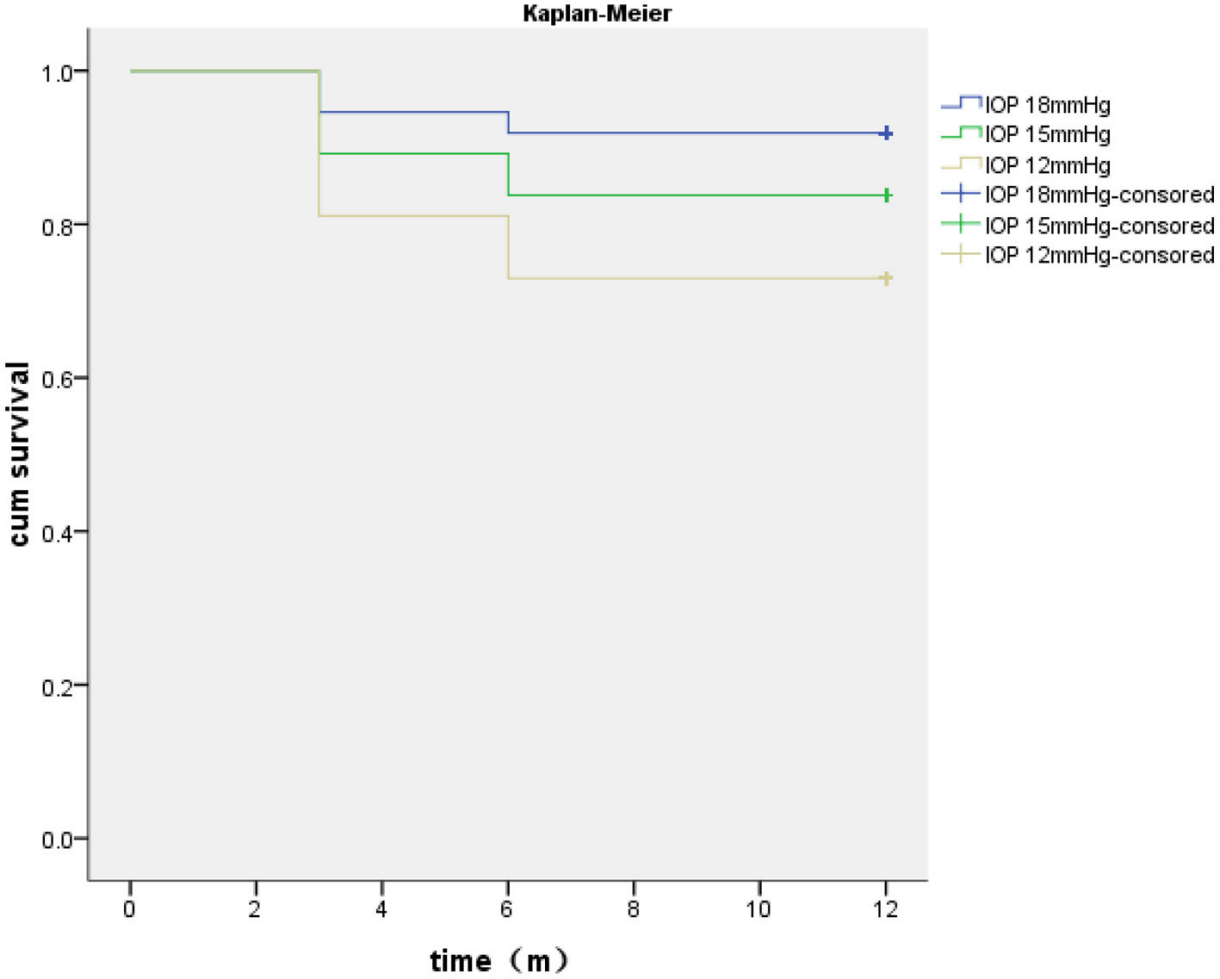
Kaplan–Meier analysis of the surgical complete success rates based on the different intraocular pressure (IOP) criteria.

The complete success rates at 3, 6, and 12 months after surgery were 97.29, 97.29, and 94.59%, respectively, according to criterion A; 97.29, 89.19, and 83.78%, respectively, according to criterion B; and 81.08, 75.68, and 72.97%, respectively, according to criterion C. The conditional success rates at 3, 6, and 12 months were 100% for standards A and B and 94.59% for standard C. The Kaplan–Meier survival curve for the complete success rates of standard A–C operations is shown in Figure 4.

## Discussion

The IOP increase in POAG patients is thought to be caused by the elevated outflow resistance of the aqueous humor at the TM adjacent and distal to the SC. Trabeculotomy can reduce the resistance in the SC and promote drainage of the aqueous humor by opening the TM and SC complex (TM, adjacent SC tissue, and inner SC wall). Thus, it can reduce the IOP in patients with POAG. In the past 10 years, circumferential trabeculotomy has been performed in adult patients with open-angle glaucoma (14). However, only a few reports have been conducted on the effectiveness of a 360° external trabeculotomy in treating adults with POAG or secondary open-angle glaucoma thus far (24). Chin et al. used a modified 360° external suture trabeculotomy to manage adult open-angle glaucoma (25). They discovered that the success rate of this surgery in POAG patients was 84% (IOP < 18 mmHg) 1 year postoperatively. The IOP decreased from 27.8 ± 12.2 to 13.1 ± 3.2 mmHg after the operation, and the average number of medications used also decreased from 2.8 to 0.5 postoperatively (25). Shi et al. performed a 1-year follow-up of 22 eyes of patients who were diagnosed with congenital glaucoma and underwent MATT (7)^.^ They discovered that the conditional success rate was 86.4% (IOP ≤ 21 mmHg). The mean IOP decreased from 33.1 ± 6.1 to 14.8 ± 2.5 mmHg after surgery, and the number of drugs the patients used to treat their condition also lessened from three kinds (range = 1–5) to none (range = 0–1) after the surgery (7).

Previous studies have shown that damage to the collector canals in adults with POAG is related to the severity of the disease and the time of onset (26–28). Race, atrophy of the collecting duct in adult patients with POAG, the disease severity, treatment duration, superior scleral venous flow wave, and ocular surface conditions may also influence the effect of SC surgery on the reduction of IOP (29). All the patients who participated in the present study had advanced POAG. The target IOP according to the AGIS was <18 mmHg. However, in a clinical study, the researchers found that the 6-year visual field mean deviation (MD) of patients was lowered by 2.5 dB when the IOP was kept below 15 mmHg; when the IOP was kept below 12.3 mmHg, the visual field remained stable. Palmberg et al. revealed that, when the IOP of patients with advanced glaucoma was controlled at 15 mmHg, the condition worsened over time in 30% of patients (30). The European Glaucoma Society recommends that the target IOP for patients with advanced-stage glaucoma should be <12 mmHg and <10 mmHg for those in terminal stages. Many studies have also shown that maintaining the IOP between 10 and 12 mmHg would be of help to control the progression of glaucoma pathology (31–34).

In view of the characteristics of patients with POAG in China (15), the filtering areas of young patients after glaucoma filtering surgery are more likely to develop scarring and require a lower target IOP (35). We performed MATT-DS-Trab on our patients, and several key points need to be emphasized regarding this procedure. Firstly, the IOP should be kept as stable as possible during the operation to avoid excessive anterior chamber bleeding. Secondly, the scleral flap should be sufficiently large (5 mm × 5 mm). The scleral pool should not only be large (4 × 4 mm) but also deep enough (the color of the choroid under the scleral bed should be visible). This is in accordance with the methodology of a study performed by Zhang et al. (15) to ensure smooth drainage and filtration of the aqueous humor. When the aqueous humor is stored in a large and deep scleral pool, it is easier to maintain IOP stability for longer periods. Thirdly, trabeculectomy should be performed as soon as possible after catheterization and trabeculotomy in order to maintain anterior chamber stability and reduce the incidence of hyphema and postoperative PAS. The scleral flap was then sutured tightly using two adjustable sutures. It is very important to remove the adjustable sutures within 4 weeks after the operation according to the IOP and the morphology of the filtration blebs. It also makes sense to use MMC during the operation and apply both topical anti-inflammatory drugs and pilocarpine during the perioperative period.

Our results showed that the mean IOP decreased to 11.43 ± 2.07 mmHg at 12 months postoperatively. We inferred that the significant decrease in IOP due to our operation might be caused by the joint promotion of aqueous humor drainage by internal and external channels, including the removal of resistance at the proximal end of the SC, leakage of the deep scleral pool, and suprachoroidal cavity. The broken end of the SC is directly exposed to the internal drainage channel of the anterior chamber, and the external drainage channel of conjunctival filtration promotes the outflow of the aqueous humor, thus reducing IOP. In addition, surgical operation proficiency and perioperative management also influence the success rate and IOP reduction.

UBM is an important method used to evaluate the correlation between aqueous humor outflow and IOP after trabeculectomy and non-penetrating deep sclerectomy (NPDS). In our study, the UBM images clearly showed all the paths under the filtering bleb and scleral flap, suggesting that aqueous filtration occurred and that the trabeculectomy and deep sclerectomy were successful. During the follow-up period, we revealed that the L-type filtering bleb was the main form at all time points, and we did not observe E-type filtering blebs. At 12 months after the operation, the F-type filtering bleb only accounted for 5.41% of blebs. Previous studies have shown that the shape of the filtering blebs is related to IOP control. Ninety-six percent of patients with ideal IOP control after undergoing deep sclerectomy developed L-shaped filtering blebs, which is consistent with the results of our study (22). We disclosed that the length and height of the scleral pool declined over time, but the difference was not statistically significant (*P* > 0.05). Zhang et al. confirmed that IOP in the stable scleral pool group was higher than that in the unstable group 12 and 24 months after CO_2_ laser-assisted deep sclerectomy surgery (CLASS) and that the difference was statistically significant (15). In other words, the stability of the scleral cistern after surgery is related to the preoperative IOP. The exact mechanism is still unclear, possibly because a high IOP before the operation causes rapid circulation of liquid after the operation, which causes a “flushing effect” and, thus, results in a more stable scleral pool. In addition, there was no significant correlation between the scleral pool size by UBM and the IOP after surgery. Similar results were determined in the studies by Zhang et al. and Jankowska-Szmul (15, 36). This indicates that the drop in IOP may not depend on the size and shape of the scleral cistern; on the contrary, the presence of the scleral cistern is very important for IOP reduction.

All of the patients enrolled in our study experienced hyphema during the operation, which is consistent with the results of previous studies (25). However, there was only a small amount of bleeding (all <3 mm), which was absorbed within 2 weeks without special treatment. In addition, Chin et al. reported that 47% of patients experienced transient high IOP after circumferential trabeculotomy (25). Some patients with an IOP >30 mmHg must receive additional medication. However, in our study, transient or persistent high IOP was not observed >2 weeks postoperatively, which may be due to the adjustable sutures in all patients during the operation, allowing doctors to regulate the IOP in a timely fashion. This could avoid damage to the optic nerve due to a high IOP or fluctuations in the IOP.

During follow-up, we measured the IOP of one patient climbing to 42 mmHg at 21 days after the operation. The ciliary body band could be observed by gonioscopy, and UBM revealed hyperechoic reflection under a filtering bleb and the scleral flap. After the suture of the scleral flap was released by laser under slit lamp, the filtering bleb was diffused and lifted and the IOP was lowered to 9 mmHg. At the last follow-up, the patient's IOP was recorded as 11 mmHg. In general, whether glaucoma surgery based on SC could work effectively depends primarily on the patency and function of the TM outflow pathway, as well as the collecting tube and its downstream passage near the incision site. This patient had advanced POAG and a C/D of 1.0, and the patient had also been taking medication for a long time before undergoing surgery. Therefore, we speculate that the function of the collecting duct and its downstream pathway may be seriously damaged. It is worth noting that, at the last follow-up, the UBM images showed that more than 90% of the filtering blebs were hypoechoic, and the results of split lamp photography revealed that the filtering blebs were diffuse and protruding. During the entire follow-up period, no thin-walled bleb-related complications were observed.

Our study has several limitations. We did not set up a control group to compare the effects of IOP reduction and the postoperative complications of other types of anti-glaucoma surgeries. The follow-up duration was also limited. Prospective, controlled, randomized multicenter studies with a larger sample size, enrollment of multiple races, and a longer follow-up period are needed to confirm the long-term efficacy and IOP reduction effect of this procedure.

## Conclusions

Microcatheter-assisted trabeculotomy combined with deep sclerectomy and trabeculectomy is based on multichannel mechanisms and has a good effect on reducing IOP 1 year after the surgery. It is safe and effective in the management of advanced POAG patients with a high success rate, but few serious complications.

## Data Availability Statement

The original contributions presented in the study are included in the article/supplementary material, further inquiries can be directed to the corresponding author/s.

## Ethics Statement

The studies involving human participants were reviewed and approved by The ethics committee of Shijiazhuang People's Hospital. The patients/participants provided their written informed consent to participate in this study. Written informed consent was obtained from the individual(s) for the publication of any potentially identifiable images or data included in this article.

## Author Contributions

All authors listed have made a substantial, direct and intellectual contribution to the work, and approved it for publication.

## Conflict of Interest

The authors declare that the research was conducted in the absence of any commercial or financial relationships that could be construed as a potential conflict of interest.

## Publisher's Note

All claims expressed in this article are solely those of the authors and do not necessarily represent those of their affiliated organizations, or those of the publisher, the editors and the reviewers. Any product that may be evaluated in this article, or claim that may be made by its manufacturer, is not guaranteed or endorsed by the publisher.
